# What Do Neighbors Tell About You: The Local Context of Cis-Regulatory Modules Complicates Prediction of Regulatory Variants

**DOI:** 10.3389/fgene.2019.01078

**Published:** 2019-10-31

**Authors:** Dmitry D. Penzar, Arsenii O. Zinkevich, Ilya E. Vorontsov, Vasily V. Sitnik, Alexander V. Favorov, Vsevolod J. Makeev, Ivan V. Kulakovskiy

**Affiliations:** ^1^Vavilov Institute of General Genetics, Russian Academy of Sciences, Moscow, Russia; ^2^Faculty of Bioengineering and Bioinformatics, Lomonosov Moscow State University, Moscow, Russia; ^3^Department of Oncology, Sidney Kimmel Comprehensive Cancer Center, The Johns Hopkins University School of Medicine, Baltimore, MD, United States; ^4^Department of Medical and Biological Physics, Moscow Institute of Physics and Technology (State University), Dolgoprudny, Russia; ^5^Engelhardt Institute of Molecular Biology, Russian Academy of Sciences, Moscow, Russia; ^6^Institute of Mathematical Problems of Biology RAS - the Branch of Keldysh Institute of Applied Mathematics of Russian Academy of Sciences, Pushchino, Russia

**Keywords:** regulatory variants, rSNP, machine learning, promoters, enhancers, saturation mutagenesis massively parallel reporter assay

## Abstract

Many problems of modern genetics and functional genomics require the assessment of functional effects of sequence variants, including gene expression changes. Machine learning is considered to be a promising approach for solving this task, but its practical applications remain a challenge due to the insufficient volume and diversity of training data. A promising source of valuable data is a saturation mutagenesis massively parallel reporter assay, which quantitatively measures changes in transcription activity caused by sequence variants. Here, we explore the computational predictions of the effects of individual single-nucleotide variants on gene transcription measured in the massively parallel reporter assays, based on the data from the recent “Regulation Saturation” Critical Assessment of Genome Interpretation challenge. We show that the estimated prediction quality strongly depends on the structure of the training and validation data. Particularly, training on the sequence segments located next to the validation data results in the “information leakage” caused by the local context. This information leakage allows reproducing the prediction quality of the best CAGI challenge submissions with a fairly simple machine learning approach, and even obtaining notably better-than-random predictions using irrelevant genomic regions. Validation scenarios preventing such information leakage dramatically reduce the measured prediction quality. The performance at independent regulatory regions entirely excluded from the training set appears to be much lower than needed for practical applications, and even the performance estimation will become reliable only in the future with richer data from multiple reporters. The source code and data are available at https://bitbucket.org/autosomeru_cagi2018/cagi2018_regsat and https://genomeinterpretation.org/content/expression-variants.

## Introduction

Recent progress in medical genetics has drawn attention to sequence variants in the regulatory regions that can alter transcription factor binding (Deplancke et al., 2016), affect cell identity (Liu et al., 2017), and bring about disorders like cancer (Killela et al., 2013) and schizophrenia (Fabbri and Serretti, 2017). Yet, the genetics approach, the genome-wide association study, is somewhat limited for it is not always able to select the causal variant from several genetically linked mutations. Genome-Wide Association Studies identify segments that usually contain many sequence variants, of which only one or a few may be directly involved in the development of a disorder. This puts forward the technologies that measure the impact of individual sequence variants directly. High-throughput functional assays (Ipe et al., 2017), such as massive parallel reporter assays (MPRA) (Kwasnieski et al., 2012; Melnikov et al., 2012; Kheradpour et al., 2013; Mogno et al., 2013; Smith et al., 2013; White et al., 2013) or alternative methods (Canver et al., 2015; Rajagopal et al., 2016) such as STARR-Seq (Arnold et al., 2013) yield direct measurements of the transcriptional response caused by sequence variants.

Despite fast progress in experimental technologies, current methods cannot test all the variants of interest at the whole genome level. The additional complication comes from the fact that a regulatory genomic variant normally affects transcription activity only in a small number of cell types (Visser et al., 2012) or in particular conditions. Thus, only a small fraction of single-nucleotide variants (SNVs) can be directly assessed for their functional effect. Generalization of the limited experimental data for more cell types or functional conditions can be achieved by computational approaches (Shi et al., 2018), e.g., the machine learning methods that can predict the estimated functional impact of the individual variants located in different regulatory elements in the human genome in silico.

The 2018 Critical Assessment of Genome Interpretation (CAGI) initiative included the “Regulation Saturation” challenge, the objective of which was to predict the functional effect of SNVs by using the data of the saturation mutagenesis MPRA (Shigaki et al., 2019). In this experiment, nine promoters and five enhancers were cloned into reporter constructs, with random changes in functional sequences introduced by the PCR-based saturation mutagenesis. The regulatory impact of particular SNVs was estimated from expression of reporters with different variants of mutated regulatory sequences.

Here, we show that the strategy of data splitting between the training and validation datasets by slicing the existing regions into several adjacent segments, which was used in the challenge, is biased by the information leakage from the local context. This information leakage allows reproducing the prediction quality of the best CAGI challenge submissions with a fairly simple machine learning approach. Finally, we discuss the actual reliability of the prediction and its possible improvement.

## Method

### Overview of the Initial Data and the Challenge Setup

The CAGI “Regulation Saturation” challenge data (Shigaki et al., 2019) included expression changes observed for more than 17 thousand induced SNVs within regulatory regions using reporters constructed from 5 human enhancers (IRF4, IRF6, MYC, SORT1, ZFAND3), and 9 promoters (F9, GP1BB, HBB, HBG, HNF4A, LDLR, MSMB, PKLR, TERT), each tested in a particular cell type (TERT was tested in two cell types). For all the regions, 25% of the data were available for model training and 75% for validation.

From the experimental data, for each SNV, two values were calculated from the gene expression change: the confidence score, i.e., the general measure of the regulatory potential, and the direction of expression change (upregulation or downregulation of the reporter expression).

According to the data providers, SNVs that significantly changed gene expression and exhibited the confidence of no less than 0.1 were considered regulatory. The expression change direction, in turn, could equal 1 or -1 for up- and downregulating SNVs, respectively, or 0 for those not passing the confidence threshold. The predictions from the CAGI challenge included both the confidence score (a regression problem) and the expression change direction (a multiclass classification problem). The average class balance between significant regulatory SNVs (confidence ≥ 0.1) and nonsignificant SNVs (confidence < 0.1) was approximately 2:7. Detailed information on the experimental data can be found in (Shigaki et al., 2019) and at the CAGI “Regulation Saturation” challenge webpage (https://genomeinterpretation.org/content/expression-variants).

As an additional validation dataset, we used the independent MPRA data on SNV expression effects for ALDOB and ECR11 regulatory regions (Patwardhan et al., 2012). The expression change P-values were capped and subjected to log-transformation to obtain confidence scores, as in the CAGI challenge data.

### Data Splitting Setup

**Figure 1:**
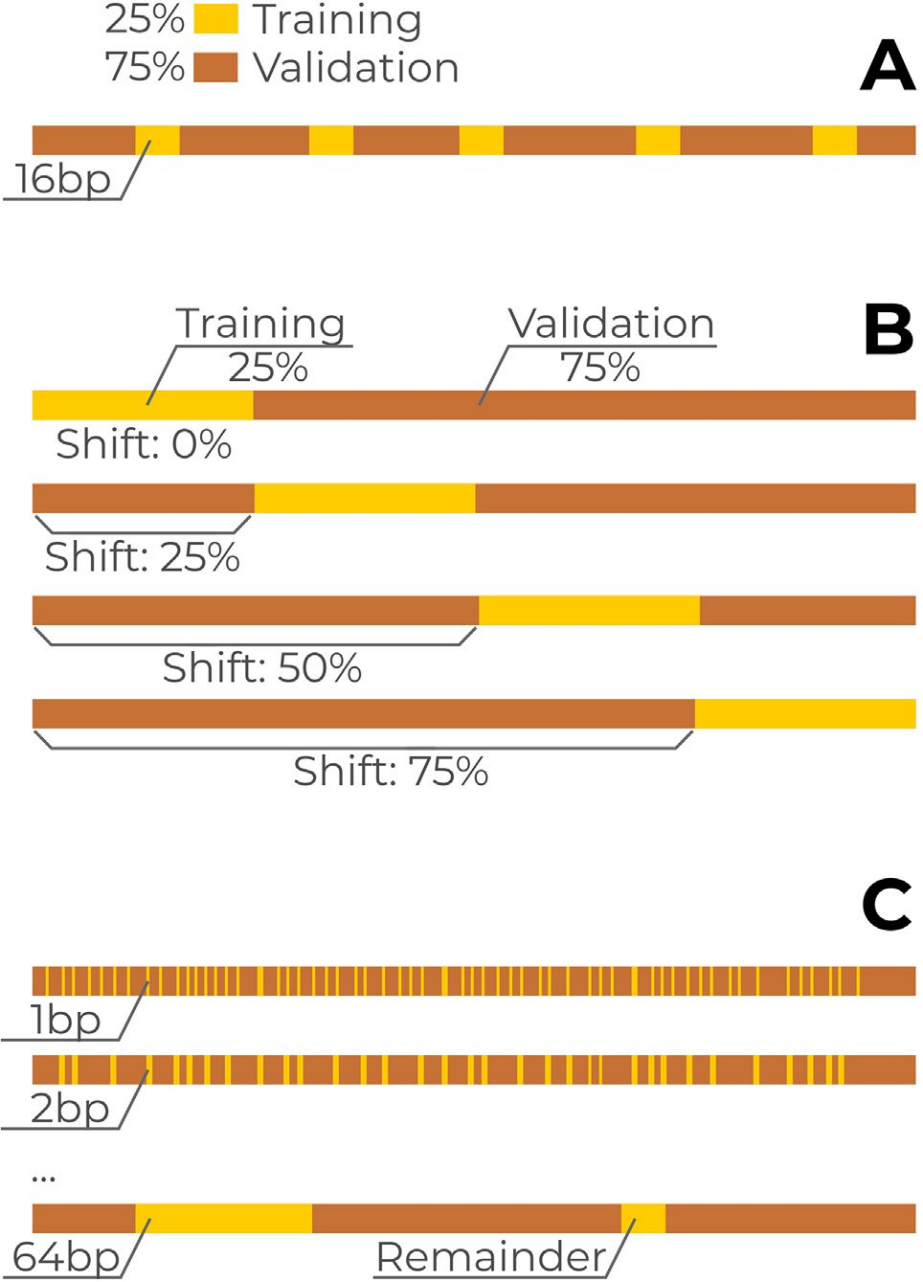
Data separation into training and validation subsets. A single reporter is shown, the scheme was identical for all reporters. Yellow bars: training subset, brown bars: validation subset. **(A)** Original CAGI setup. For each reporter, the training subset of single-nucleotide variants (SNVs) (25% from total) consists of multiple 16bp blocks spanning over neighboring reporter coordinates. **(B)** Continuous blocks covering 25% of reporter length for each reporter with a varying shift from the reporter 5’ end. **(C)** Training data with varying block lengths from 1 to 64bps.

The initial data splitting strategy of the CAGI challenge was the following. Approximately 25% of the SNVs from each reporter were allocated for training and 75% for model validation. The training data were arranged as a series of segments alternating with validation data and more or less distributed uniformly along each reporter with the total volume proportional to the reporter length. For each reporter, the training data were distributed into multiple 16 base pair long blocks with spacers in between that were used as the validation dataset (**Figure 1A**). For each particular training block, the confidence score and the direction of the expression change were provided in each position for each of the three possible nucleotide substitutions. The complete data for all the regulatory regions were made publicly available after the challenge. On these data, we used the two additional train/validation splitting setups in our analysis. First, we used a single continuous block per reporter with the block length equal to 25% of the corresponding reporter length (**Figure 1B**). Second, we varied block lengths from 1 to 64 base pairs (**Figure 1C**) and sampled the blocks from each reporter in a uniform manner, maintaining the 25/75 ratio between the training and validation data.

### Prediction Quality Measures

The challenge setup and existing submitted predictions allowed us to assess the prediction performance of both confidence scores (continuous scale from 0 to 1, the regression problem) and expression change directions (-1, 0, or 1, the three-class classification problem). We computed several prediction quality measures.

Quality measures for the confidence score prediction (the regression problem): Mean absolute error (MAE_c_), mean squared error (MSE_c_), mean error (ME_c_), Pearson (PCC_c_), and Spearman (SCC_c_) correlation coefficients between the predicted and experimental confidence scores. Quality measures for the expression change direction prediction (the multiclass classification problem): Pearson (PCC_d_) and Spearman (SCC_d_) correlation coefficients between the predicted and experimental expression change direction values.

We also performed binary classification (regulatory SNVs versus neutral SNVs, i.e., combined 1 and -1 classes versus 0 class) by setting discrimination thresholds for predicted confidence scores (i.e., solutions of the regression problem). We assumed that the regulatory SNVs of 1 and -1 classes should receive higher predicted confidence values, while the neutral SNVs of the 0 class (no significant expression change) should receive lower predicted confidence values. We computed the areas under the curves for the receiver operating characteristic (AUCROC) and precision-recall curve (AUPRC) for this binary classification and used these quantities as primary measures of prediction quality throughout the study.

### Machine Learning Framework

For the primary analysis, we used a standard software implementation of a nontweaked random forest (Boulesteix et al., 2012) from the scikit-learn package (Pedregosa et al., 2011). With 500 estimators, the other parameters were set to default values. As we split the data only into two parts, the training and validation datasets, we did not perform any hyperparameter tuning to avoid overfitting (Cawley and Talbot, 2010). Possible redundancy of the features should have no significant effect on the random forest classifier. Three different data sources were used for feature generation: sequence motif analysis, functional genomics data, and neuron values from the last layer of the DeepSea (Zhou and Troyanskaya, 2015), the deep learning-based algorithm for predicting the chromatin effects of sequence alterations at single-nucleotide resolution.

#### Features Derived From Sequence Motif Analysis

2,168 features were generated by applying PERFECTOS-APE (Vorontsov et al., 2015) for all SNVs in each reporter. PERFECTOS-APE estimates the impact of nucleotide substitutions within transcription factor binding motif occurrences, which are relevant in terms of putative regulatory effects. We used mononucleotide and dinucleotide (771 + 313) position weight matrices from HOCOMOCO collection v11 representing binding motifs of human transcription factors (Kulakovskiy et al., 2018). Two floating-point values were obtained and log-transformed for each SNV: P-value of the best binding motif occurrence overlapping the reference allele, and P-value fold change for the alternative allele. Conversion from position weight matrix scores to motif P-values was performed with precomputed thresholds-to-P-value dictionaries available in HOCOMOCO.

#### Features Derived From Genomic Tracks

5,857 features were generated from genomic tracks representing different types of experimental data: ChIP-Seq, ATAC-Seq, and DNase-Seq, providing information regarding transcription factor binding and open chromatin regions in different cell types. Genomic coordinates of reporters that were used for feature extraction are provided in **Supplementary Table 1**. UCSC liftOver (Meyer et al., 2013) was used to convert coordinates between hg19 (CAGI default, DNase-Seq data) and hg38 (ATAC-Seq, ChIP-Seq) genome assemblies. The floating-point values were linearly scaled to fit the [0,1] interval using scikit-learn MinMaxScaler separately for each reporter. For each genomic feature and each reporter, mean nonnormalized values were saved and used as additional 5,857 features (constant for all SNVs in each reporter).

##### GTRD Chip-Seq-Based Features

5,703 features were generated from the ChIP-Seq read alignments obtained from the Gene Transcription Regulation Database (GTRD) (Yevshin et al., 2017; Yevshin et al., 2018). The ChIP-Seq alignments were processed in a uniform manner: the reads were extended to 300 bp, and the extended read coverage at each genomic position (i.e., for each SNV) was calculated using DeepTools (Ramírez et al., 2014).

##### Features Generated From ENCODE Dnase-Seq and ATAC-Seq Data

106 features were generated from 53 consolidated DNase accessibility profiles provided by the Roadmap Epigenomics project (Kundaje et al., 2015). The features were extracted from the signal (bigwig) profiles of fold change and p-value tracks. Similarly, 48 features were generated from ENCODE (Dunham et al., 2012) ATAC-Seq fold change signal profiles (bigwig).

#### Features Derived From the Deepsea Neural Network

DeepSEA is an artificial neural network trained to predict the results of more than 900 ENCODE experiments for each position in the human genome from sequences of 1000-nucleotide windows surrounding the target genomic positions. DeepSEA is considered to learn and integrate transcription factor binding motifs, including composite elements from transcription factor interactions, and other aspects of the regulatory grammar. While direct predictions of DeepSEA do not allow proper classification of MRPA results, DeepSEA provides useful features that notably improve the next layer of the machine learning application. 4,595 features were generated by the DeepSEA (Zhou and Troyanskaya, 2015). We used 919 neuron outputs of the last DeepSEA layer for the reference allele, 919 outputs for the alternative allele, 919 values of differences between neuron outputs for the reference and alternative alleles, as well as E-values and log fold changes of the difference.

#### Unrelated Features From Nonrelevant Genomic Regions

To roughly estimate the contribution of information leak from genomic segments of training data to neighboring validation segments, we made a set of irrelevant DeepSEA features extracted from alien genomic regions using the same training/validation layout. There were no reporters on chromosome 3, thus we generated the irrelevant features by extracting chr3 data using the reporter coordinates on the correct chromosomes. The only exception was IRF6 reporter, which was located at the chromosome 1 (which is longer than chromosome 3) and out of chr3 expansion, so we performed modulo operation with chr3 length as the second argument. The exact coordinates are provided in **Supplementary Table 1**. By construction, the resulting features carry no actual biological information from the target reporters.

#### Source Code and Features Data Availability

The source code and features data are available at the BitBucket repository (https://bitbucket.org/autosomeru_cagi2018/cagi2018_regsat). The actual data from the saturation mutagenesis massively parallel reporter assays are available at the CAGI challenge website (https://genomeinterpretation.org/content/expression-variants).

## Results

### Deepsea Provides Sufficient and Necessary Features for the Top-Performing Solution

**Figure 2:**
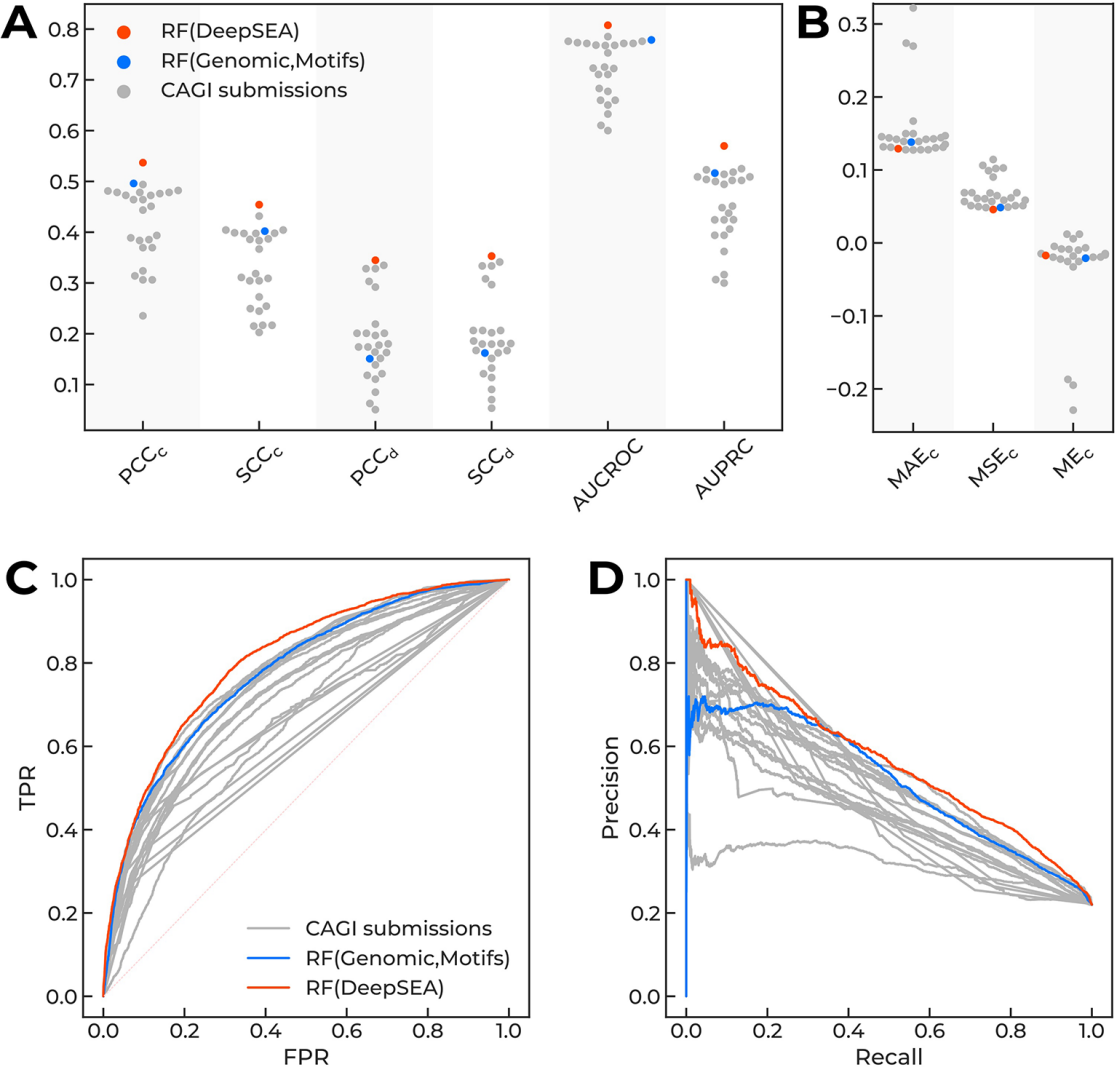
The performance of different models predicting regulatory single-nucleotide variant (SNVs) of the CAGI “Regulation Saturation” challenge. Orange dots, Random Forest classifier using DeepSEA features. Blue dots, Random Forest classifier using features based on genomic data and sequence motif analysis. Grey dots, CAGI challenge submissions. **(A**, **B)** Different performance measures for prediction of expression direction (d) and confidence scores (c): Pearson (PCC_c_ and PCC_d_) and Spearman (SCC_c_ and SCC_d_) correlation coefficients, area under curve for receiver operating characteristic (AUCROC), area under precision-recall curve (AUPRC), mean absolute error (MAE_c_), mean squared error (MSE_c_), and mean error (ME_c_). **(C**, **D)** Receiver operating characteristics and precision-recall curves.

Several top-performing submissions for the CAGI challenge (Shigaki et al., 2019) used DeepSEA (Zhou and Troyanskaya, 2015) to compute the features for classifiers based on decision trees. To clarify the contribution of DeepSEA features to the top-level predictions of variant effects in the original CAGI setup, we compared the performance of newly built random forest classifiers with CAGI submissions. Diverse performance measures showed a very consistent result: the random forest model atop the DeepSEA-only features was the top-performing solution without any hyperparameter tweaking (**Figure 2**, **Supplementary Table 2**).

We did not perform any comprehensive feature selection, but checked a few fixed combinations of features sets (**Supplementary Table 2**) and used two classifiers as the baseline for the further analyses: the top-performing DeepSEA-based approach, RF(DeepSEA), and the classifier based on genomic features and sequence motif analysis, RF(Genomic,Motifs), which demonstrated good but not the top-level prediction performance. Of note, DeepSEA-processed features were essential and the only necessary to reach the top performance level.

### Prediction Performance Achievable for a Reporter Strongly Depends on the Training Data From the Same Reporter

**Figure 3:**
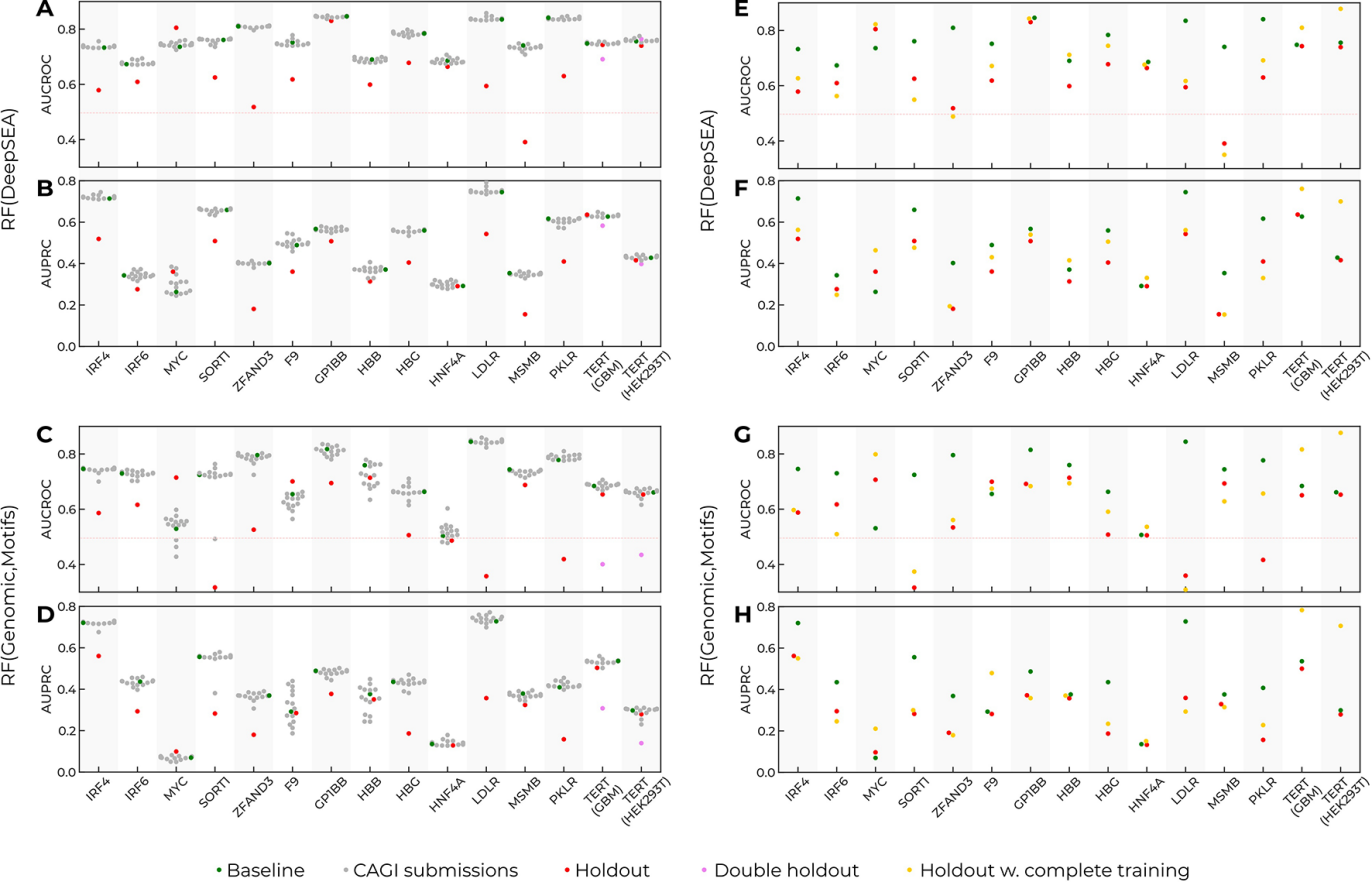
Prediction performance drops as the data from target reporters are excluded from training. Training on the complete data improves prediction quality, but cannot compensate for the holdout of the data for single-nucleotide variants (SNVs) from the target reporter. Green dots, the baseline models trained in the CAGI setup. Grey dots, the CAGI submissions. Red dots, the models trained in the CAGI setup with the data from the target reporter held out. Violet dots, the performance for the TERT target reporter with the data from both TERT assays held out from training. Yellow dots, the models trained with the complete data from all reporters excluding the target reporter. Reporter names are given at the X-axes. **(A**, **B)** Area under precision-recall curve (AUPRC) and area under curve for receiver operating characteristic (AUCROC) for Random Forest with DeepSEA features (baseline and holdout models). **(C**, **D)** AUPRC and AUCROC for Random Forest with Genomic signal and sequence motif features (baseline and holdout models). **(E**, **F)** AUPRC and AUCROC for Random Forest with DeepSEA features (baseline, holdout, complete training models). **(G**, **H)** AUPRC and AUCROC for Random Forest with Genomic signal and sequence motif features (baseline, holdout, complete training models).

In the original challenge setup, the training data included fragments of data from each reporter (see Method, **Figure 1**). Thus, predictions for a particular reporter used the data from known regions of the same reporter. To investigate to what extent the training data setup contributed to the model performance, we made up a “holdout model” for each reporter. The holdout model for a particular target reporter used all the data from the baseline model for training but did not include the SNVs from the target reporter (**Figures 3A–D**). Next, we estimated the resulting performance of such models for each target reporter and realized that the performance of the holdout models drastically degraded for all reporters as compared to the baseline models. There were two exceptions worth mentioning.

First, the MYC reporter did not follow the global trend. This reporter contained only a few regulatory SNVs (86 of 1790), thus leading to unstable AUCROC/AUPRC values. Second, the prediction for TERT reporters did not change upon a single holdout. Indeed, data for TERT were obtained in two cell types, and holdout of data from a single cell type could be compensated for by the information on the same sequence tested in the second cell type. Holding out both cell types resulted in reduced model performance similar to that for the other reporters with the target constructions held out.

To verify that the performance reduction was not caused by the reduced training data size, we used the complete data from all reporters for training (rather than 25% as in the original setup), excluding the target reporter. On average, the increased training data volume slightly improved the prediction quality (**Figures 3E–H**) but did not compensate for the effects from holding out the data from the target reporter. Thus, the inclusion of SNVs from the target reporter was critical for the models’ performance.

These results agree well with the data from dimensionality reduction by UMAP (McInnes et al., 2018) shown in **Supplementary Figure 1**, where regulatory and non-regulatory SNVs are indistinguishable, but SNVs belonging to particular reporters form clear well-defined areas. Particularly, SNVs of the MYC reporter form two separate clusters, which might be related to its special behavior in holdout tests.

### Assessing the Contribution of Local Context

There can be two alternative explanations of the major contribution of the training data from the target reporter into the prediction performance. Known SNVs from the target reporter may either allow prioritizing the features relevant to a particular reporter or provide an information leakage due to the fact that regulatory function is performed by a sequence segment rather than an isolated nucleotide, and such segment has characteristic composition of k-mers (Mariño-Ramírez et al., 2004), e.g. corresponding to a local arrangement of transcription factor binding sites.

The original CAGI setup of the training-validation data (**Figure 1A**) provides “sneak peeks” at different regions of each reporter, thus possibly revealing key regulatory sites in the surrounding sequence. To validate this hypothesis, we used continuous blocks of 25% of the length of each reporter to construct a new training dataset (**Figure 1B**) and placed the blocks at 0–75% from the 5’ end of each reporter, with the remaining data used for validation. For each reporter, we then computed the difference in AUCROC and AUPRC between the new model and the baseline solution. The holdout model was taken as a reference.

**Figure 4:**
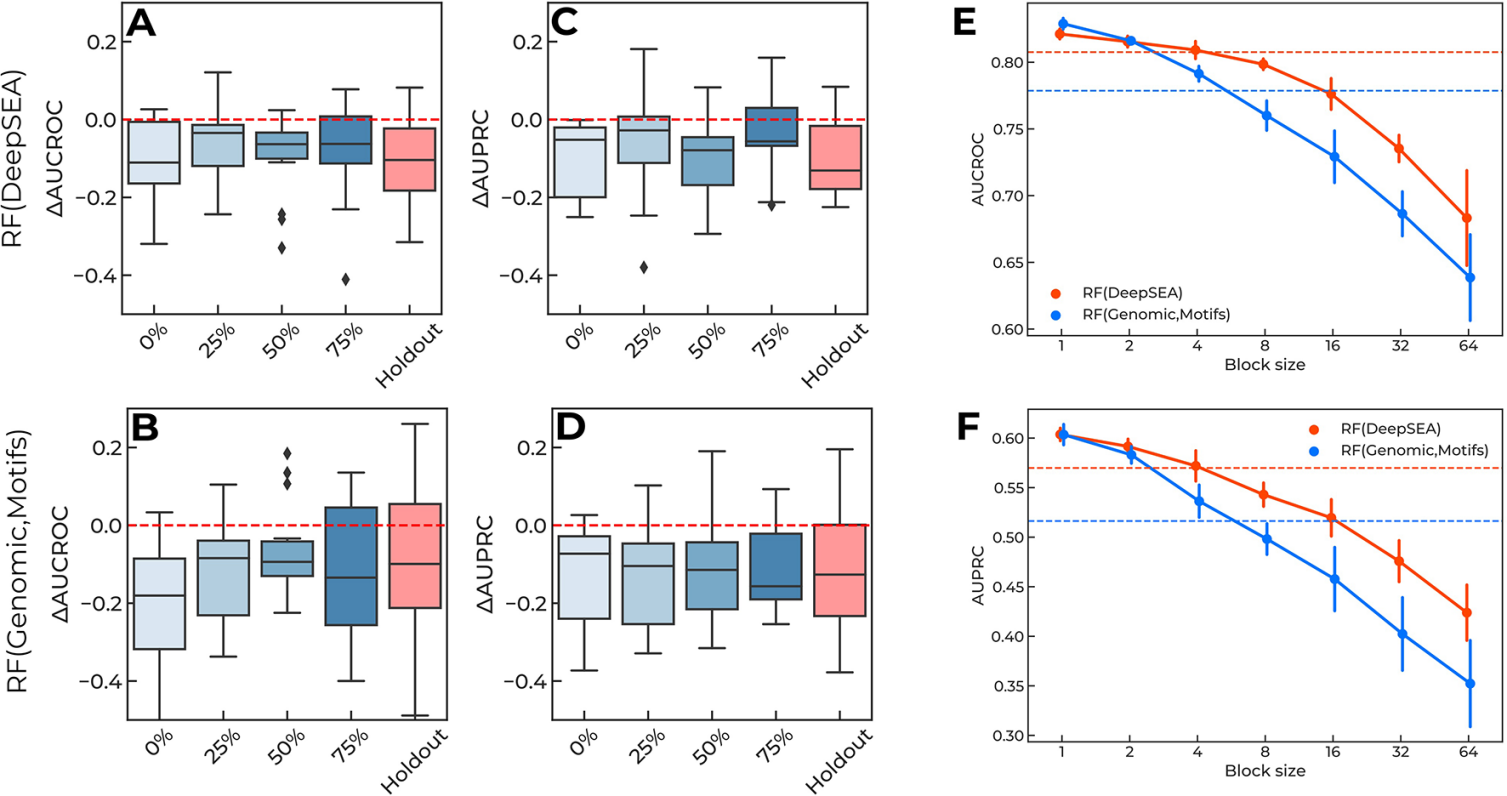
**(A-D)** Training data of a single continuous block per reporter degrade performance of the prediction regulatory single-nucleotide variants (SNVs). X-axis, locations of training data blocks relative to the 5' ends of the reporters. Y-axis, the difference in AUCROC and AUPRC values for each model versus the baseline. The holdout of SNVs from each reporter is shown for the reference. Boxplots aggregate data from all reporters. Random Forest using DeepSEA features: **(A)** AUCROC, **(C)** AUPRC. Random Forest using genomic data and sequence motif features: **(B)** AUCROC, **(D)** AUPRC. **(E**–**F)** Shorter blocks in training data improve models performance due to information leakage. Orange lines: Random Forest classifier using DeepSEA features. Blue lines: Random Forest classifier using features based on genomic data and sequence motif analysis. Solid lines show the mean and standard deviation of 10 random samples with a fixed block length (X-axes). Dashed lines show the values reached in the original CAGI setup of the training data. **(E)** AUCROC values, **(F)** AUPRC values.

The results are presented in **Figure 4**: the single-block design of training data degraded the performance of the models to the “holdout” level. Importantly, the relative placement of the training data block (i.e., the shift from the 5’ end of each reporter measured as the percentage of the reporter length, **Figure 1B**) did not affect the overall quality reduction. Furthermore, by varying the block size (**Figure 1C**) and maintaining the training data size (i.e., by composing the required volume of training data from multiple fixed-size non-overlapping blocks at random positions), we observed that shorter blocks resulted in higher model performance (**Figures 4E, F**), probably due to a more uniform sampling of each reporter. Notably, the original CAGI setup used blocks of 16bps. In the case of training with random blocks sampling, we observed a similar performance for shorter blocks of 4bps. We believe this may come from the specific regular sampling of blocks and spacers in the original CAGI setup, where the spacers between the blocks were selected in a non-random regular fashion providing pieces of information from all regions of each reporter.

All in all, this confirms that there is an information leakage from known regulatory SNVs included in the training data, which is related to the local context of the regulatory regions.

### Interdependencies of Neighboring Reporter Segments Allow for Good Predictions With Irrelevant Features

The grammar of regulatory regions makes neighboring positions in regulatory regions dependent, e.g., if they are localized within a particular transcription factor binding site. This is also reflected in the distribution of regulatory SNVs along reporters, with many SNVs with significant expression effects being clustered together (Shigaki et al., 2019). Thus, a locally correlated feature may allow predicting SNV effects given the training data are evenly distributed within a particular reporter, as in the original CAGI setup. That is, any nonlocal feature used in the setup, in which the mutual information between the training and validation data is not negligible, will have a great chance to induce information leakage.

To validate this hypothesis, we used irrelevant features extracted from the single chromosome (chr3) for all reporters (see Methods). This procedure removes any meaningful information conveyed by features but retains local intercorrelation of feature values for neighboring variants. One could expect a huge reduction in performance, comparable to that of a “random guess” classifier. However, Random Forest atop of irrelevant features performed quite well reaching AUCROC of 0.75 and AUPRС of 0.47 (**Supplementary Figure 2**).

To estimate the practical usability of models directly trained on CAGI MRPA data, we used independent data on SNVs tested in ALDOB/ECR11 reporters (Patwardhan et al., 2012). Two baseline solutions, RF(DeepSEA) and RF(Genomic,Motifs), trained either on the CAGI “blocks” setup and or on the complete CAGI data, were used to predict SNV effects in the independent validation dataset.

The results appear to be rather discouraging (**Table 1**), with the DeepSEA-based solution performing poorly if trained on the CAGI blocks setup and even worse if the complete CAGI data was taken. The model based on genomic signals and motif analysis showed moderately better performance, improving its results on ALDOB/ECR11 validation dataset with the increased training data amount. However, in holdout tests, this model displayed generally weaker performance than that of DeepSEA solution, thus complicating the choice of the most reliable approach, since the validation dataset contained data for only two reporters. A general solution may appear with increased volume of experimental data, e.g. with multiple reporters tested in a single cell type in a single experiment, thus allowing direct and more robust cross-validation.

**Table 1:** Area under precision-recall curve (AUPRC) and area under curve for receiver operating characteristic (AUCROC) reached by the baseline models on the independent validation dataset.

Model	AUCROC	AUPRC
RF(DeepSEA), CAGI setup training	0.6	0.2
RF(DeepSEA), Complete training	0.5	0.15
RF(Genomic,Motifs), CAGI setup training	0.67	0.2
RF(Genomic,Motifs), Complete training	0.79	0.31

## Discussion

Assessment of a regulatory effect is an essential step in the annotation of SNVs, important both for understanding deeper functional consequences of somatic mutations (Kalender Atak et al., 2017) and SNP allele effects. Computational approaches to functional annotation require training data sets, and MRPAs could become one of the key data sources. MPRA and machine learning are two recent technologies, the power of which is yet to be harnessed for the progress of genetic studies, particularly in regulatory genomics. MPRA is an expensive method, and only a limited number of variants or genomic regions can be studied in an experiment directly. Open challenges like CAGI can facilitate the application of machine learning techniques and suggest the most fruitful setup for the design of MPRA experiments to obtain the most suitable information for further reliable predictions.

In this study, we have confirmed that (1) the features preprocessed with machine learning methods (DeepSEA) provide a better basis for prediction of MPRA data than simple genomic signals and statistical quantities (motifs), and (2) the local context of selected regulatory regions has a significant contribution to MPRA-based training and validation, which apply strong constraints on possible training/validation layouts.

Eukaryotic regulatory regions are adapted to harbor diverse combinations of transcription factors under different conditions, bringing about complex statistical interactions between regulatory genetic variants and other features of the loci (Greenside et al., 2018) that affect machine learning applications. At this point, it is difficult to completely estimate the biological nature and scale of local grammar restrictions.

Surprisingly, the separation of the train and test data remains an open question in machine learning applications for genomics and sometimes might result in overoptimistic estimates of the prediction quality. A model with a large number of parameters could behave unexpectedly, indirectly deducing genomic coordinates from the given features and using only the data on the test loci, instead of employing genome-wide data for the prediction (Schreiber et al., 2019). In a recent notable publication (Xi and Beer, 2018), it was demonstrated that high performance of a model predicting enhancer-promoter interaction (Whalen et al., 2016) was explained by information leakage caused by inappropriate split of training and validation data.

By adopting the DeepSEA preprocessing, we estimated the extent to which the results of MPRA-based training could be used after allowing for the local grammar. The results might look disappointing, because the prediction appears to be significantly inflated due to the information leakage from the training SNVs in the neighboring segments. However, after holding out the data on a particular reporter, it is still possible to obtain better-than-random predictions.

If a model achieves good prediction performance only for the train regions, with the actual experimental information, the practical value of such prediction is somewhat limited. Particularly, annotation of somatic mutations and eQTLs requires the predictions at genomic regions, in which prior information is unavailable. Thus, it is practically important to estimate the degree, to which MRPA-trained models are suitable for prediction of effects within non-tested regions.

Unfortunately, the small number of reporters in the study and high variability of the prediction quality between the reporters did not allow us to obtain a stable measure of the prediction accuracy. Particularly, the prediction for the MRPA dataset from another study, although performed at a small number of reporters, displayed contrasting results, in which the features based on simple statistical quantities outperformed the features based on DeepSEA preprocessing.

The organizers of the CAGI challenge have spent a notable effort to provide a proper evaluation of computational predictions by slicing the data from tested reporters in a zebra-like fashion between training and validation subsets. However, as we demonstrate here, this layout is not a sufficient counter-measure against the strong local interdependencies within particular regulatory regions, thus allowing the models to benefit from the information leakage. Training data of single blocks from each reporter (**Figure 4**) could be a better alternative in terms of models evaluation, but also might be overly restrictive in terms of diversity of training data. A more reliable way would be to test multiple reporters in a single cell type and to use the complete data from the independent reporters for training and evaluation.

To sum up, we believe that further careful exploration of MRPA-based training data setups is necessary as soon as more MRPA data would be accumulated and new powerful machine learning methods would be adopted. This would lead to improved stacking of machine learning models and allow better predictions in non-explored genomic regions.

## Data Availability Statement

Publicly available datasets were analyzed in this study. These data can be found here: https://bitbucket.org/autosomeru_cagi2018/cagi2018_regsat, https://genomeinterpretation.org/content/expression-variants.

## Author Contributions

DP, AZ, IV, VS, and AF performed data analysis. IK and VM supervised the study. All the authors have participated in the manuscript preparation.

## Funding

This study was supported by the Russian Foundation for Basic Research grants 18-34-20024 and 19-29-04131, Skoltech Systems Biology Fellowship (to IV), Program “Postgenomic technologies and perspective solutions in the biomedicine” of the RAS Presidium, project АААА-А19-119091090024-4, and Russian Program of Fundamental Research for State Academies. The CAGI experiment coordination was supported by NIH U41 HG007446 and the CAGI conference by NIH R13 HG006650.

## Conflict of Interest

The authors declare that the research was conducted in the absence of any commercial or financial relationships that could be construed as a potential conflict of interest.
