# Supplementary material for: What Do Neighbors Tell About You: The Local Context of Cis-Regulatory Modules Complicates Prediction of Regulatory Variants
Source: Front Genet. 2019 Oct 31;10:1078. doi: 10.3389/fgene.2019.01078 (PMC6834773; doi:10.3389/fgene.2019.01078)
Supplement: Supplementary file 1 [file Image_1.pdf]

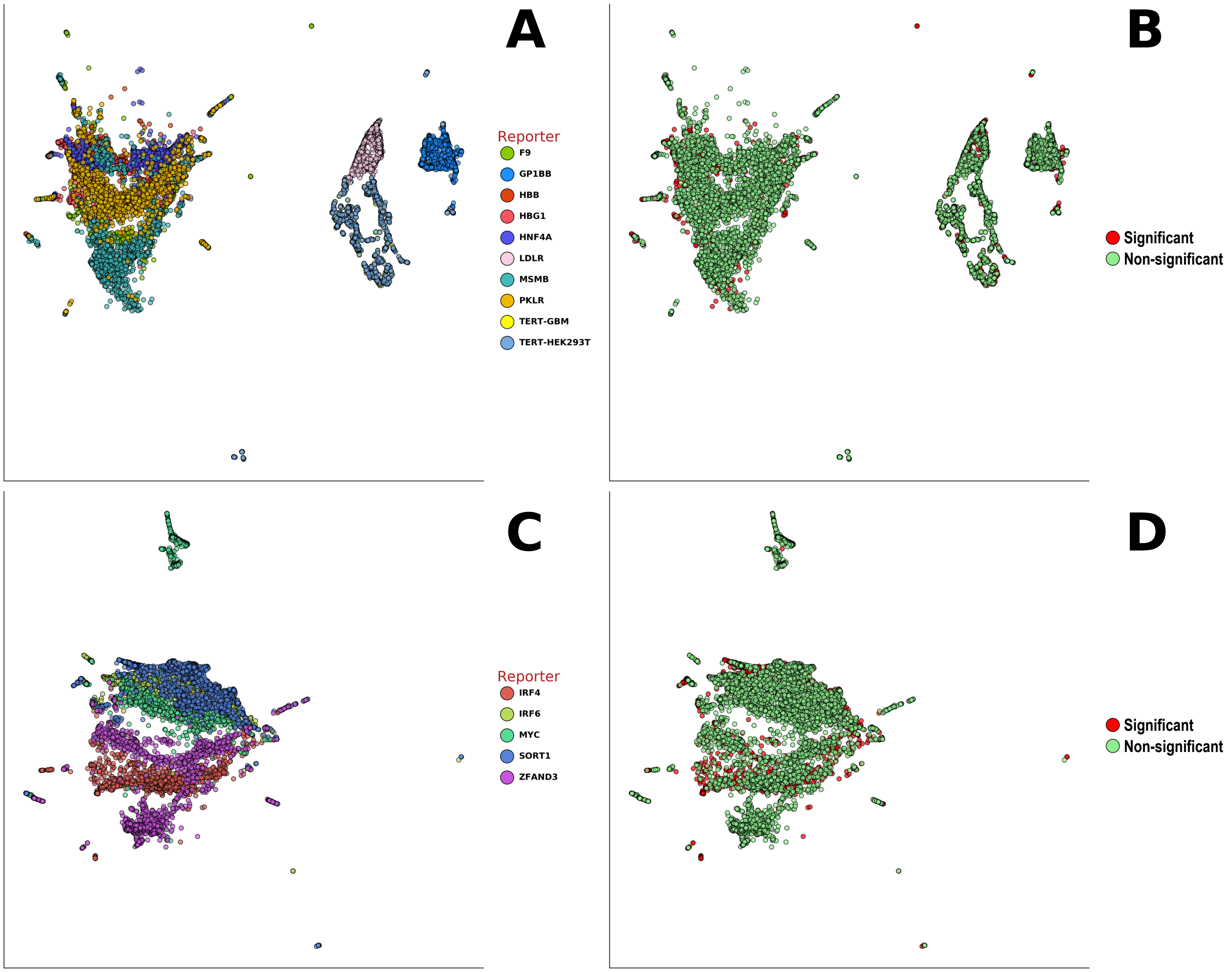

Supplementary Figure 1. SNVs in promoters (A, B) and in enhancers (C, D) in the two-dimensional UMAP space. The plots illustrate clear separation of reporters, while significant regulatory SNVs are non-trivially mixed with the non-significant SNVs. The SNVs of the MYC reporter are arranged in two distant clusters, which is probably linked to its unusual behavior in holdout tests.
