# Supplementary material for: What Do Neighbors Tell About You: The Local Context of Cis-Regulatory Modules Complicates Prediction of Regulatory Variants
Source: Front Genet. 2019 Oct 31;10:1078. doi: 10.3389/fgene.2019.01078 (PMC6834773; doi:10.3389/fgene.2019.01078)
Supplement: Supplementary file 2 [file Image_2.pdf]

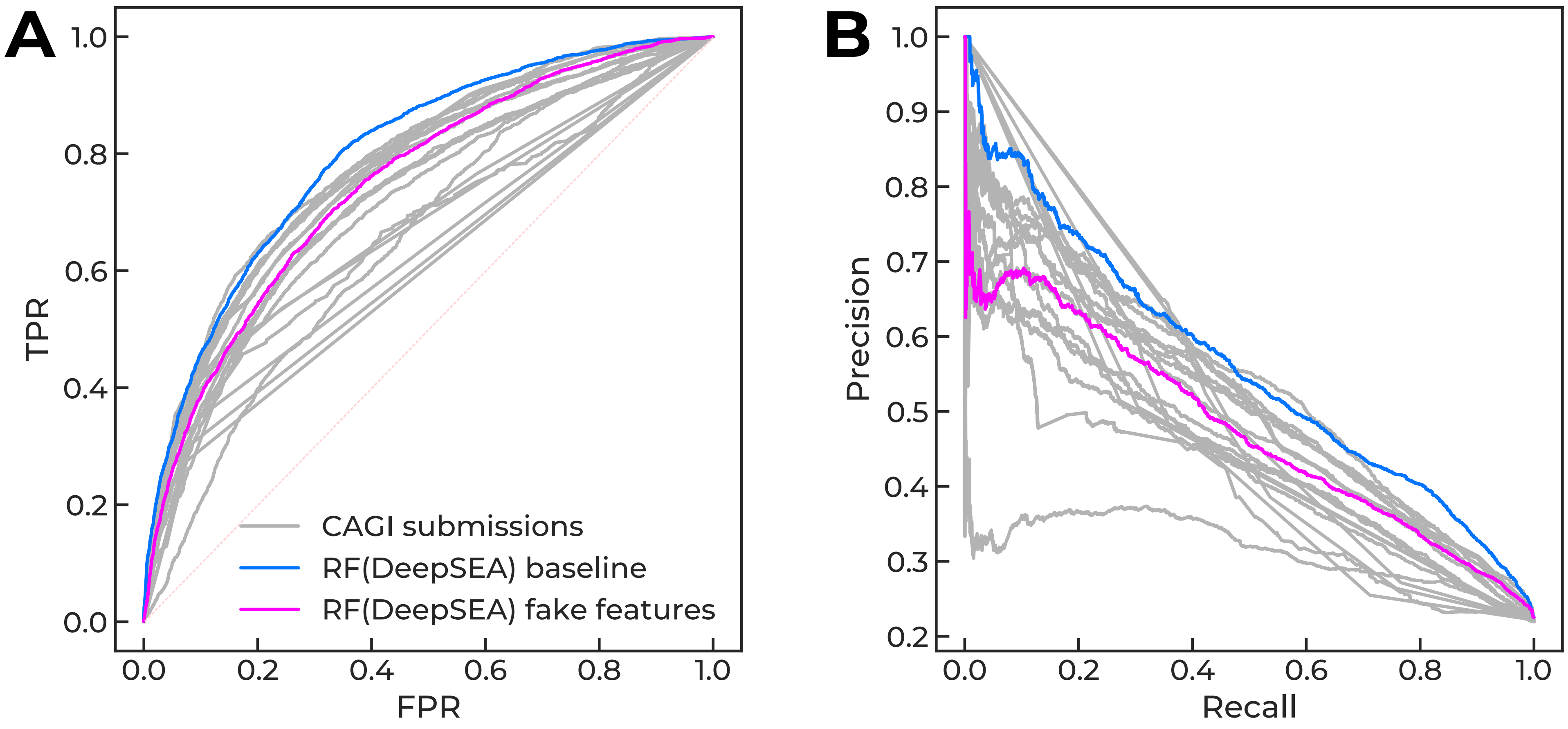

Supplementary Figure 2. Non-relevant features allow reaching good prediction quality.
(A) Receiver operating characteristics, (B) Precision-recall curves.

Blue, the baseline model (Random Forest atop real DeepSEA features);
Magenta, the irrelevant baseline model
(Random Forest atop alien DeepSEA features from non-relevant genomic regions of chr3);
Grey, the CAGI submissions.
